# Replace, amplify, transform: a qualitative study of how postgraduate trainees and supervisors experience and use telehealth for instruction in ambulatory patient care

**DOI:** 10.1186/s12909-022-03175-3

**Published:** 2022-02-22

**Authors:** Hannah L. Anderson, Joshua Kurtz, Daniel C. West, Dorene F. Balmer

**Affiliations:** 1 Children’s Hospital of Philadelphia, 3401 Civic Center Blvd. 9 NW 76, Philadelphia, PA 19104 USA; 2 Department of Pediatrics, Perelman School of Medicine at the University of Pennsylvania, Philadelphia, USA

**Keywords:** Telehealth, Telemedicine, Educational technology, Postgraduate medical education

## Abstract

**Background:**

Little is known about using telehealth patient visits as an educational mode. Therefore, rapid implementation of telehealth during the COVID-19 pandemic had to be done without understanding how to optimize telehealth for education. With the likely sustained/post-pandemic use of telehealth in ambulatory patient care, filling gaps in our understanding of how telehealth can be used for instruction in this context is critical. This study sought to understand perceptions of pediatric postgraduate trainees and supervisors on the use of telehealth for instruction in ambulatory settings with the goal of identifying effective ways to enhance learning during telehealth visits.

**Methods:**

In May–June of 2020, the authors purposefully sampled first- and third-year postgraduate trainees and supervising attendings from pediatric fellowship programs at one institution that implemented telehealth for instructional activities. They conducted semi-structured interviews; interviews lasted a median of 51 min (trainees) and 41 min (supervisors). They conducted interviews and data analysis iteratively until reaching saturation. Using thematic analysis, they created codes and constructed themes from coded data. They organized themes using the Replace-Amplify-Transform (RAT) model, which proposes that technology can replace in-person learning and/or amplify and transform learning.

**Results:**

First-year trainees (*n* = 6), third-year trainees (*n* = 5) and supervisors (n = 6) initially used telehealth to replace in-person learning. However, skills that could be practiced in telehealth visits differed from in-person visits and instructional activities felt rushed or awkward. Trainees and supervisors adapted and used telehealth to amplify learning by enhancing observation and autonomy. They also transformed learning, using telehealth to develop novel skills.

**Conclusions:**

To harness telehealth for instructional activities, our findings indicated that trainees and supervisors should shift from using it as a direct replacement for in-person education to taking advantage of novel opportunities to amplify and transform education in PGME. The authors provide data-driven recommendations to help PGME trainees, supervisors and educators capitalize on the educational advantages of telehealth.

**Supplementary Information:**

The online version contains supplementary material available at 10.1186/s12909-022-03175-3.

## Background

As a result of the COVID-19 pandemic in Spring 2020, health systems exponentially increased the use of telehealth visits as a safe alternative to in-person ambulatory patient visits [1–6]. Although this shift from in-person to telehealth visits allowed for continued delivery of many aspects of ambulatory patient care, it abruptly changed clinical training in postgraduate medical education (PGME) [7–9]. Opportunities for traditional clinical instruction, in which trainees learned through supervised practice, in person teaching, and feedback, were lost [8]. Instead, PGME trainees remotely joined their supervisors in virtual settings and telehealth became a primary way of conducting instructional activities [7, 8].

Advantages of telehealth in providing ambulatory health care, including ease and efficiency for patients, families, and providers, have been reported [10]. These advantages suggest that telehealth will remain a fixture of ambulatory care rather than an artifact of the COVID-19 pandemic [5, 6]. There is a large body of research about online learning [11] and curricula to teach telehealth skills [12, 13], but the advantages (and disadvantages) of the use of telehealth for experiential clinical instruction in PGME are largely unknown. With the likely sustained use of telehealth post-pandemic, filling gaps in our understanding of how telehealth can be used for instruction in ambulatory patient care is critical to provide a foundation for future instructional activities in PGME [14].

As a step toward filling this gap, we sought to understand PGME trainee and supervisor perceptions of using telehealth for instruction in ambulatory settings. We included the perspectives of both trainees and supervisors because recent studies of the rapid changes in PGME due to the COVID-19 pandemic indicate differences between trainee and supervisor perspectives on the use of technology in education [15–18]. Our central research question was, “How do PGME trainees and supervisors in pediatrics use and experience telehealth for education in pediatric ambulatory patient care?” We sought to use these findings to identify strategies to help trainees, supervisors and health professions educators capitalize on the educational advantages and mitigate educational disadvantages of telehealth [19, 20].

## Methods

### Study design and sample

We conducted this qualitative interview study at an urban academic children’s hospital, employing thematic analysis as our methodological orientation [21]. Seeking participants with sufficient exposure to telehealth, we purposefully sampled postgraduate fellowship trainees (hereafter referred to as PGME trainees) from six pediatric fellowship training programs that rapidly converted to telehealth ambulatory visits. PGME training in the United States typically consists of 3 years of general pediatric post-graduate training followed by 3–4 years of additional postgraduate training for those interested in pediatric subspecialty careers. We interviewed individuals in year 1 and year 3 of subspecialty training (year 4 and 6 of total postgraduate training, respectively). We chose to sample these PGME trainees to understand any differences in experiences between trainees with little subspecialty training experience (1 year of training) and significant experience (3 years of training).

In May–June 2020, we recruited, via email, all PGME trainees in the above-mentioned programs (41 PGME trainees total). Consistent with our qualitative research design, we collected and analyzed data iteratively; we ended data collection when we had sufficient data to answer our targeted research question and were not identifying new concepts in incoming interviews [22]. We interviewed trainees on a rolling basis as they agreed to participate and reached sufficiency after interviewing 11 PGME trainees. Interviews had a median length of 51 min. Interviews ranged from 38 to 69 min.

In the interviews with PGME trainees, we asked trainees to identify supervisors who had supervised them in telehealth visits and recruited those supervisors by email. Like the PGME trainee interviews, we ended data collection when we had sufficient data to address our research questions and were not identifying new concepts in the data. We reached sufficient data after 6 supervisor interviews. Interviews had a median length of 41 min; interviews ranged in length from 26 to 58 min. All trainees and supervisors who agreed to participate were interviewed; no participants dropped out or were excluded from the study. The Children’s Hospital of Philadelphia Human Subjects Review Committee determined that the study was exempt from review. We obtained verbal consent from all participants. We report all qualitative methodology considerations for the study using the COREQ List (Supplement).

### Interviews and data analysis

Three authors (HA, DB, DW) developed one interview guide for PGME trainees and one for supervisors (Supplementary material: Appendices A and B). Interview guides were designed to elicit discussion about a study subject’s preparation for, use of, and experience with the use of telehealth for instruction in ambulatory patient care. Interview guides were piloted with one volunteer PGME trainee and one volunteer supervisor from recruited programs; pilot interviews were not included in the final data set. One author (HA) conducted all interviews, which were recorded and transcribed using Ava Scribe and de-identified by HA. We managed data in ATLAS.ti.

We collected and analyzed data from interviews with PGME trainees and supervisors in parallel. Two authors (HA, JK) created initial inductive codes (words that act as labels for important concepts in the data) after reviewing the first five transcripts. A third coder (DB) reviewed and critiqued initial codes. Three authors (HA, JK and DB) revised codes based on incoming data until they reached consensus on a final list of codes after 12 of the 18 interviews. As analysis progressed, HA and JK created themes by scrutinizing patterns in coded data. We adopted a thematic analysis approach that allows for two different level of themes: *manifest* themes (patterns explicitly in the data) and *latent* themes (higher-order patterns of ideas in the data) [21]. The group of authors discussed themes over the course of 5 months and reached consensus about the veracity of four *manifest* themes: 1) observation and feedback was altered in the context of telehealth, 2) trainees’ autonomy could be affected by telehealth, 3) trainees had different exposure to clinical skills and content areas compared to in-person learning, and 4) both trainees and supervisors adapted to technological barriers and/or advantages over time.

**Fig. 1:**
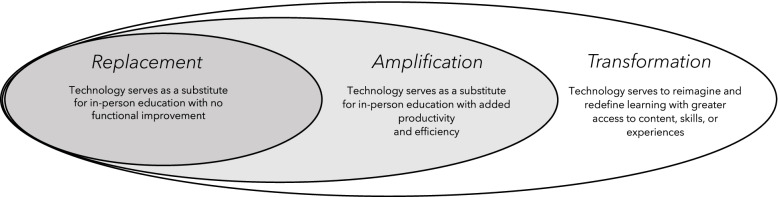
The Replace-Amplify-Transform Model (Adapted from Hughes, 2006 and McHugh, 2014)

To better understand our findings in the context of educational technology, two authors (HA and JK) analyzed manifest themes and organized higher-order *latent* themes in the data using a model from the educational technology literature. The Replace-Amplify-Transform (RAT) model proposes that technology tools can serve one of three purposes: (a) as a direct replacement for traditional learning activities (e.g., simply takes the place of another activity); (b) as a way to amplify learning (e.g., make an educational activity more efficient or impactful); and (c) as a means to transform learning (e.g., result in new types of learning not previously possible) (see Fig. 1) [23].

## Results

### General findings

Rising second-year and graduating third-year subspecialty PGME trainees endorsed similar uses and experiences regarding telehealth, therefore we do not distinguish between these two groups of trainees (completed 1 vs 3 years of training). Similarly, we did not appreciate differences by PGME training program. Thus, we report findings from the collective groups of trainees and supervisors and attribute illustrative quotes to role. A summary of PGME trainee and supervisor characteristics is included in Table 1.

**Table 1:** Participant characteristics from a qualitative study of postgraduate medical education trainees (*n* = 11) and supervisors/attending physicians (*n* = 6) at an urban academic pediatric hospital

PGME Training Program	Rising 2nd year PGME trainees	Graduating 3rd year PGME trainees	Supervisors of PGME trainees
Adolescent Medicine	1	–	1
Cardiology	–	2	2
Developmental/Behavioral Pediatrics	2	–	1
Hematology/Oncology	1	1	–
Gastroenterology	1	1	1
Rheumatology	1	1	1
**Total:**	**11**		**6**

All PGME training programs offered guidance on how to use telehealth technology to conduct a patient care visit but did not offer guidance or training on how to leverage telehealth for education during the visit. Prior training or experience in telehealth technology seemed to increase familiarity and comfort level with navigating telehealth technology, particularly for supervisors. Regardless of prior experience or training, both PGME trainees and supervisors perceived that telehealth, in and of itself, was neither ‘good’ nor ‘bad’ for education. Rather, the educational advantage or disadvantage depended on how telehealth was used for instruction.

PGME trainees and supervisors consistently spoke about using telehealth to replace, amplify, and transform education but to varying degrees. Overall, trainees spoke about amplifying and transforming education more frequently than supervisors. In the following sections, we expand on these general findings and provide examples of how trainees and supervisors experienced and used telehealth. We organized these component findings by higher-order, latent themes that were derived from RAT: replace, amplify, and transform.

### Component findings by replace-amplify-transform

#### Telehealth as a replacement for education in in-person ambulatory settings

Early in the shift to telehealth, PGME trainees and supervisors used telehealth as a direct substitute for learning in in-person ambulatory settings. In other words, they attempted to keep the telehealth experience as close as possible to the in-person experience. However, both trainees and supervisors expressed frustration with telehealth-as-replacement because the knowledge and skills gained in telehealth visits were inevitably different from the knowledge and skills gained in in-person visits. They reported that completing certain aspects of a physical exam was not possible over telehealth, and patients with certain diagnoses could not be seen via telehealth. For example, children who were followed long-term for congenital heart disease could be seen in a telehealth visit but children who were newly referred for evaluation for a heart murmur could not. Moreover,

*“While I don't think every kid needs an extensive physical exam, I do think that there are pieces of learning about physical exam that you lose. – Trainee #5*

According to PGME trainees and supervisors, telehealth reduced opportunities for dialogue and precepting. Trainees struggled to ask questions, such as questions to clarify patient care or about how to improve their performance. They were unsure of when they should sit back and watch a telehealth visit versus take the lead on learning how to construct a plan of care.

*“In terms of learning you lose out on a lot… You're coming up with a plan on the fly and hoping it’s okay. And so even if there's a good opportunity for precepting, even if it is a straightforward case, there's just not back and forth conversation.” – Trainee #7*

Supervisors shared trainee’s concerns about the infrequent opportunities for precepting. Supervisors struggled with the awkwardness of virtual communication, reduced ability to read social cues, and difficulty in recognizing teachable moments, all of which hampered instruction:

*“Reading the temperature in the room and those social skills… that's been the hardest part. My [teaching] style is, a pointer here, a pointer there. So when do I chime in?” – Supervisor #3*

#### Telehealth as an amplification of education in in-person ambulatory settings

PGME trainees and supervisors were prompted by the limited of replacement to use telehealth to amplify learning by leveraging its features that added value to clinical care. For instance, supervisors leveraged presumed barriers to promote trainees’ autonomy. Although telehealth technology can typically only show one provider at a time, supervisors used this “feature” to let trainees operate as the primary provider in a telehealth visit. Families were less prone to look to the attending when the trainee was speaking:

*“One of the hard things about precepting a fellow in the office in an in-person visit is when the fellow’s trying to talk and you see the family's always looking at you as the attending, right? They can't do that on Telehealth…It does give you an opportunity to let the fellow just go ahead and take over” – Supervisor #5*

Supervisors noted that PGME trainees learned specific telehealth exam skills, such as how to interpret visual findings through a screen. Supervisors took advantage of telehealth visits to observe trainee’s history-taking skills, something they did not do in inpatient visits:

“*It is such an opportunity to observe them in a way that we don’t normally do… To talk about their history taking skills, which is something we rarely observe in fellows anymore. So I think it's a golden opportunity in that regard.” – Supervisor #6*

Trainees reported learning how to clearly communicate instructions to patients and families via telehealth. For instance, they learned to re-think the kinds of questions they asked in visits and use clearer language:

“*The questions we ask our patients can be very subtle and confusing, and even offensive at times. I personally rely a lot on gestures, like I'm reading their body language… and that guides my questioning. Without that to rely on, I've had to think a lot more about language and being very clear about what I'm asking.” – Trainee #2*

Some trainees saw the telehealth as a novel opportunity to practice relationship-building with patients and families:

“*So much of our fellowship, in addition to clinical learning, is learning how to engage and support our patients… [telehealth] is a really wonderful, unique way to do that that we never would have gotten exposure to...” – Trainee #1*

PGME trainees and supervisors remarked on the capacity of telehealth to afford insight into patients’ homes. They recognized that how families used home medical equipment and asked patients to demonstrate home health care and medication regimens was a unique source of knowledge:

“ *… having families actually bring their physical pill bottle to the camera to look and see, make sure labels are correct. I think is a really interesting addition to the visit.” – Trainee #8*

#### Telehealth as a transformation of education in in-person ambulatory settings

Some PGME trainees and supervisors actively sought ways to use telehealth to transform education; that is, they used telehealth to redefine or reconfigure teaching and learning. They learned new skills like telehealth triage, which entailed identifying complaints and types of patients appropriate for telehealth versus in-person or emergency room visits.

“*It definitely gave me practice triaging. I would run it by attendings, and they would be coaching me, like ‘did you see this? You said we could see them in two months, but if we do that we could also see them late and miss this part of it’ that I wasn’t thinking about.” – Trainee #10*

PGME trainees sometimes reconfigured their learning by improving time management and advanced preparation for visits. They reached out to supervisors with questions prior to visits, rather than waiting for lags in the daily schedule to seek feedback as they often did in-person. For example, they used pre-clinic huddles to maximize time in a telehealth visit:

*“[I] ask the [supervisor], could you meet for 20 minutes before your clinic starts to just run through patients? So we'll go through each one, big picture, and go through things we’re worried about… it's much more efficient and then in that period you can also say like okay, why would you ask that? And what would you do? To get some of that modeling and have a learning interaction even before you see the patient.” – Trainee #3*

Without an in-person physical exam, PGME trainees relied heavily on history-taking and interviewing skills in order to create a treatment plan. Some capitalized on this experience with telehealth not just to amplify but to take their history-taking abilities to a new level.

*“As far as clinical skills, I think really honing in on history taking skills and preparation … it’s almost like survivor mode where you're given a set of limited resources and you have to independently figure out how to get by, so yes, I’m learning to remain vigilant for red flags” – Trainee #7*

Via telehealth, PGME trainees were exposed to new types of patients, e.g., patients in long-term care facilities or patients with multiple/severe disabilities that made travel to in-person visits unfeasible. Trainees were also exposed to health disparities, particularly disparities in patients’ home and social environments:

“*I'm learning how different it is to hear about someone's home environment versus seeing them in their home environment and how much that changes how you understand …I think that it provides a really interesting context to their normal lives that we don't normally see.” – Trainee #3*

Trainees spoke about how telehealth prompted them to question traditional ways of learning and practicing medicine. Some trainees re-thought their testing procedures; others questioned what ‘counts’ as an exam and how often in-person exams were really required.

*“It definitely makes you think, what are the things that can be done locally? Was that study absolutely needed? And is it absolutely needed now?” – Trainee #8*

Supervisors also remarked on the possibilities of redefining observation and feedback. Some thought that feedback sessions integrated into telehealth were more systematic and prioritized than frequent, more casual in-person feedback encounters:

*“It does change [feedback]. You have to make more of a concerted effort because sometimes when you're walking out of the room, it's easy to say, you handled that really well. Or, I would have said this. Or, did you notice how they reacted when you said this? So I do think that it takes more effort… You're more systematic about doing it. You prioritize it more.” – Supervisor #1*

## Discussion

In this qualitative study, PGME trainees and supervisors initially used telehealth visits as a direct replacement for teaching and learning in in-person pediatric ambulatory settings. However, replacing in-person education with telehealth had limitations; certain types of patients could not be seen, and certain aspects of the physical exam could not be performed virtually. The use of telehealth also disrupted spontaneous and private moments of teaching and real-time feedback.

Based on our data, using telehealth as a direct replacement for in-person ambulatory patient care experiences did not fully support learning. Frustrated by replacement, PGME trainees and supervisors reported that telehealth visits could amplify or even transform education in pediatric ambulatory settings. Our findings suggest that telehealth can provide opportunities for trainees to hone skills such as history-taking, learn new skills such as telehealth triage, and better prepare for visits. For supervisors, telehealth can help make feedback more systematic.

That PGME trainees and supervisors used telehealth to amplify and transform instructional activities can inform other aspects of training in PGME. Some have raised concerns that opportunities for trainee autonomy and independent decision-making may be diminished in telehealth visits [24]. However, we found that supervisors in this study embraced the features of telehealth that amplified trainee autonomy by allowing trainees to independently conduct visits. Furthermore, feedback was hampered when trainees and supervisors were operating from a replacement mindset. However, supervisors transformed their feedback processes when they used telehealth to directly observe trainees’ skills and systematically provide feedback on what they observed.

We found that PGME trainees and supervisors in this study all received technical instruction from their programs prior to their telehealth experiences, however, we recognize that lack of technical skills or aversion with technology, reported pre-COVID-19 [25], may have contributed to the limitations that participants reported in using telehealth as a replacement. As telehealth continues to be used, this aversion may decrease. Trainees and supervisors may develop skills or comfort with telehealth technology that allows them to optimize telehealth for instruction by finding opportunities for amplification and transformation that we did not capture in this study [26–28].

Based on our findings, we developed list of data-driven recommendations, or practice points, for using telehealth to amplify and transform education in ambulatory settings (see Table 2). Our recommendations are organized according to the educational advantage it affords and could be used by others seeking to more effectively use telehealth to support learning in PGME. Beyond implications for practice, we found that the Replace-Amplify-Transform model is a useful lens for understanding how telehealth is used for instruction in the health professions. Telehealth-as-replacement can compromise possibilities, creativity, efficiency, and actually thwart education. Telehealth-as-amplification or transformation affords novel opportunities for teaching and learning and may be usefully applied to health professions education evaluation or research [29].

**Table 2:** Educational advantages and practice points for telehealth recommended for postgraduate trainees/fellows and supervisors/attending physicians

	Educational advantage	Practice Points for telehealth
***Amplification***	*Autonomy*	-Encourage supervisors to step back and allow trainees to run telehealth visits
	*Exposure*	-Highlight learning opportunities in difficult to teach concepts, such as social determinants of health -Encourage trainees to practice less frequently observed skills, such as history-taking
	*Preparation*	-Provide education-specific training for trainees and supervisors (not just technology training) -Pay attention to patient populations and consider how trainees’ learning on various topics/diagnoses may need to be supplemented
***Transformation***	*Huddles*	-Implement pre-clinic huddles that center instructional activities -Ensure huddles are scheduled with ample time for questions and spontaneous teaching
	*Triage*	-Involve trainees in telehealth triage decisions to allow for experience in decision-making
	*Feedback*	-Observe and give feedback on key skillsets, such as history-taking -Schedule time for feedback on key skillsets

### Limitations

This study was exploratory and conducted in one medical specialty and at one institution; thus, it may have limited transferability to other specialties and institutions. However, many of the instructional activities in ambulatory-based clinical education are common across specialties and institutions. Data were collected retrospectively; recall bias may have influenced responses from the participants in our study may not represent all of the ways PGME trainees and supervisors learned to use telehealth over time. However, our iterative data collection and analysis allowed for some adjustments as participants became more familiar with telehealth over the course of the study.

## Conclusions

To harness telehealth for instructional activities, our findings suggest that PGME trainees and supervisors should use telehealth to amplify and transform education in PGME, rather than as a replacement for in-person instruction. Our findings and data-driven recommendations can help PGME trainees, supervisors and educators capitalize on the educational advantages of telehealth and serve as a useful starting point for medical educators seeking to integrate telehealth into PGME training.

## Supplementary Information

**Additional file 1: Appendix A.** Interview Guide for Postgraduate Fellows: Main Questions. **Appendix B.** Interview Guide for Supervisors: Main Questions.

**Additional file 2.**

## Data Availability

The qualitative datasets generated and/or analyzed during the current study are not publicly available due to institutional data-sharing policy but are available from the corresponding author on reasonable request.

## Abbreviations

PGME: Postgraduate medical education
RAT: Replace-Amplify-Transform model of educational technology
