# Supplementary material for: Replace, amplify, transform: a qualitative study of how postgraduate trainees and supervisors experience and use telehealth for instruction in ambulatory patient care
Source: BMC Med Educ. 2022 Feb 22;22:118. doi: 10.1186/s12909-022-03175-3 (PMC8861601; doi:10.1186/s12909-022-03175-3)
Supplement: Supplementary file 1 — Additional file 1: Appendix A. Interview Guide for Postgraduate Fellows: Main Questions. Appendix B. Interview Guide for Supervisors: Main Questions. [file 12909_2022_3175_MOESM1_ESM.docx]

**Appendix A: Interview Guide for Postgraduate Fellows: Main Questions**

- 1. What is your fellowship training program?
  2. What year are you in your fellowship training?
  3. Think back to your last telehealth patient encounter. Can you walk me through that encounter?
     1. What knowledge, skills or attitudes did you take away from that?
  4. What did you do, if anything, to influence/advance your learning via tele-health, if anything?
  5. Thinking about telemedicine visits compared to seeing patients in person with your supervisor in clinic, what aspects of your learning that were quite similar? What aspects were quite different?
  6. Thinking about your telehealth experiences as a whole, what should we keep/eliminate/change to improve PGME?
  7. Imagine I’m a 1^st^ year fellow, just getting started and just getting introduced to telehealth… What advice would you give to help me maximize my learning from telehealth?

**Appendix B: Interview Guide for Supervisors: Main Questions**

- 1. What is your fellowship training program?
  2. Think back to your last telehealth patient encounter. Can you walk me through that encounter?
  3. In your division, who or what prepared you for teaching (or facilitated teaching) via tele-health, if anything?
  4. Do you have a sense of how fellows were prepared for learning via tele-health?
  5. Do you have any sense that some fellows are more inclined to “take charge” of their learning? How does this play out in the setting of telehealth?
  6. Thinking about telehealth visits compared to in person, what aspects of your teaching were quite similar? What aspects were different?
  7. What aspects of assessment were quite similar? What aspects were different?
  8. Thinking about your telehealth experiences as a whole, what should we keep/eliminate/change to improve PGME?
